# It is the flash which appears, the movement will follow: Investigating the relation between spatial attention and obstacle avoidance

**DOI:** 10.3758/s13423-015-0821-8

**Published:** 2015-05-16

**Authors:** Rudmer Menger, H. Chris Dijkerman, Stefan Van der Stigchel

**Affiliations:** Experimental Psychology, Helmholtz Institute, Utrecht University, Heidelberglaan 1, 3584 CS Utrecht, The Netherlands

**Keywords:** Obstacle avoidance, Human, Reaching, Planning, Visuomotor control

## Abstract

Obstacles are represented in the attentional landscape. However, it is currently unclear what the exclusive contribution of attention is to the avoidance response. This is because in earlier obstacle avoidance designs, it was impossible to disentangle an effect of attention from the changing features of the obstacle (e.g., its identity, size, or orientation). Conversely, any feature manipulation could be interpreted as an attentional as well as an obstacle effect on avoidance behavior. We tested the possible tuning of avoidance responses by a spatial cue in two experiments. In both experiments, spatial and nonspatial cues were separately given as go cues for an obstacle avoidance task. Participants had to reach past two obstacles in Experiment 1, and past a single obstacle in Experiment 2. We found that when the right obstacle was flashed, participants veered away more and produced more-variable trajectories over trials than in conditions with nonspatial and left spatial cues, regardless of the presence or absence of another obstacle. Therefore, we concluded that the tuning of avoidance responses can be influenced by spatial cues. Moreover, we speculated that a flashed obstacle receives more attentional weighting in the attentional landscape and prompts a stronger repulsion away from the obstacle.

According to Baldauf and Deubel (2010), the planning of a complex movement entails the creation of a so-called “attentional landscape” that weights the attentional distribution across all action-relevant locations in the visual layout of the workspace. In addition to locations, the weighting is sensitive to motor-related aspects of the task, such as the required accuracy, with more weight being attributed to a location that requires more accuracy. Movements are then executed toward the (highest) peak in the landscape and away from low(er) activity regions or valleys. Cisek (2007) has further argued that the peaks in attentional landscapes instantiated by action-relevant objects are simultaneously parallel motor plans for interacting with objects. For instance, Wood et al. (2011) have shown that visual salience dominates early visuomotor competition in reaching behavior. These researchers manipulated the salience of potential targets using varying degrees of luminance and found that participants directed their early reach trajectories toward more luminant—and therefore salient—targets in an array of multiple potential targets.

Other experiments have shown that the capture of attention by a nontarget can change the motor responses to target objects (Chang & Abrams, 2004; Tipper, Howard, & Jackson, 1997; Welsh, 2011; Welsh & Elliott, 2004; Welsh, Elliott, & Weeks, 1999). However, these nontarget objects were not obstructing, and sometimes were not even physical objects at all. So, although these studies may shed light on the effect of distracting stimuli on motor behavior, we cannot be certain that the results for attentional manipulations in obstacle avoidance would be the same. Indeed, the changes in motor responses evoked by salient distractors and visual cues are thought to be due to the biased resolution of competition between a target and distractor during action selection (i.e., which object to reach for), whereas obstacle features (e.g., location, size) must be incorporated in the motor plan that brings the hand and arm around the obstacle and toward the target. After all, in obstacle avoidance, the goal is *not* to interact with the obstacle. This means that a separate motor plan to interact with an obstacle is redundant (once the target is identified). Instead, the detected obstacle information should be incorporated into the motor plan toward the target. This means that an obstacle does not act as a distinct attractor of motor activity, but rather as a *repeller* of motor activity.

Given that obstacles are represented in the attentional landscape, it remains to be seen what the exclusive contribution of attention itself is to the avoidance response. In earlier experiments, attentional manipulations had co-occurred with changing features of the obstacle (e.g., its color similarity to the target; Menger, Dijkerman, & Van der Stigchel, 2013), or feature manipulations of the obstacle co-occurred with attentional changes. Now, for the first time, the present experiments offer the opportunity to study the effect of attention without changing the spatial features of the to-be-avoided obstacles.

In the present experiments, we studied the relation between attentional capture and obstacle avoidance. As such, we had participants perform reaches for and evasions of physical objects. Furthermore, we manipulated attentional capture by flashing LEDs embedded within the obstacles as a cue for movement onset. We expected that the tuning of the avoidance response could be influenced by a spatial cue. The spatial cues were given by flashing two LEDs on a single obstacle on one side of the workspace. We compared this to a baseline cue in which an LED was flashed on both obstacles present in the workspace. We confirmed the nonspatial nature of this cue in Experiment 2 by comparing it with a no-flash condition. Moreover, we hypothesized that the capture of spatial attention immediately prior to obstacle avoidance would shift the trajectories farther away from the obstacles. This directionality of repulsion—for example, a right-obstacle flash leading to more deviation away from the right obstacle—was checked in Experiment 2 with a single-obstacle setup.

## Method

### Participants

Ten participants (seven women, three men) volunteered via informed consent for Experiment 1, and another ten (six women, four men) for Experiment 2. They participated in this study in exchange for curricular credit. All participants were right-handed, had normal or corrected-to-normal vision, and were naïve as to the purpose of the study. The faculty’s institutional review board under the Medical Research Act issued a formal written waiver that this research project did not require approval from a Medical Ethics Review Committee.

The sample size was determined using power analysis software, namely G*Power (Franz Paul, Universität Kiel, Germany). We obtained a partial eta-squared (*η*_p_^2^) value from an earlier study (Menger, Van der Stigchel, & Dijkerman, 2012). The effect size, *f*, was determined to be .57. This related to the difference in deviations of the hand movements between the target-with-similar-obstacle condition and the target-with-dissimilar-obstacle condition (i.e., a main effect of target–distractor similarity on the deviation of the hand from the obstacle). The effect size of .57 would be detected with a precision *α* = .05 (two-sided) and with *β* = .05 (power = 95%).

## Apparatus and stimuli

**Fig. 1:**
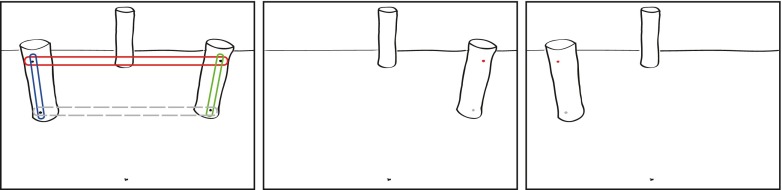
View of the layout of the experimental workspaces from a participant’s perspective. The left panel shows the dual-obstacle setup, whereas the middle and right panels show the two single-obstacle setups. In the left panel, colored ovals show the configurations of LED stimulation: “top” stimulation, “right” stimulation (spatial), “left” stimulation (spatial), and “bottom” stimulation (catch trials). The starting location of the hand (black cross) was at (0, 0), and the front point (for the participant) of the target was at (0, 400). The left obstacle’s inside point was located at (–100, 200), and the right obstacle’s inside point was located at (100, 200).

Participants were seated behind a table with an unmarked workspace (450 × 300 mm). Within the workspace, four elements were present: the target object, two nontarget objects, and the starting button (see also Fig. 1). The target object stood on a trigger that responded when the target was removed. Tall, cylindrical objects (5.5 cm × 15 cm) were used as the targets and nontargets. The target was placed at 400 mm depth and 0 mm width with reference to the starting location, whereas the nontargets were placed at 200 mm depth and at ±100 mm width. Two red-colored LEDs were embedded (near the top and the bottom) within the left and right nontargets and faced toward the participant. The LEDs were programmed to emit bursts of light for 30 ms.

The movement kinematics were recorded using MiniBird magnetic markers (Ascension) at a sampling rate of 100 Hz over 3 s. The markers were attached to the tips of the participants’ index fingers and thumbs (see also Mon-Williams & McIntosh, 2000). Furthermore, by securing the cables to the participants’ arms and hands, great care was taken to avoid interference by the markers themselves with the movements.

### Design

For each condition in Experiment 1, two LEDs were always flashed simultaneously. The location of the LED flashes was varied across four conditions: top, left, right, and bottom (see also Fig. 1). Trials in the bottom condition served as no-go catch trials (20%), and those in the other conditions served as go trials. There was no difference in the required responses in these go conditions.

Experiment 1 consisted of 108 trials: 30 repetitions of each experimental condition (top, left, and right) and 18 catch trials. The trials were randomized such that the first half of the repetitions of each condition took place in the first half of Experiment 1, and the second half of the repetitions in the latter half of Experiment 1. The experimental trials were preceded by 16 practice trials (four repetitions of each condition).

In the additional Experiment 2, three conditions were tested in three batches: top, bottom, and no LED stimulation, in dual, single-left, and single-right nontarget setups (see Fig. 1). A sound was played as a go cue, while the location of the simultaneous flash informed the participant of the required response: Top flashes and no flashes counted as the go trials, whereas bottom flashes counted as no-go (catch) trials. All other details were identical to those of Experiment 1.

### Procedure

Participants were mid-sagittally aligned with the center of the workspace. They were instructed to rest their right thumb and index finger on the starting button in a closed pincer posture until task execution was required. The experiments were self-paced: Once participants had pushed the starting button, a random interval between 800 and 1,200 ms elapsed before any LED(s) would flash. Depending on the condition, the flash(es) could indicate the go signal or the no-go signal. When a no-go condition was presented, the participants were not allowed to move, and after a short delay (3 s) they were allowed to proceed with the next trial. When a go condition was presented, participants were instructed to smoothly and rapidly reach for the target object with their right hand. Their goal was to lift the target from the table and place it back with the same hand. We further instructed participants to grasp the middle of the target object with their thumb and index finger.

## Dependent measures and analysis

All analyses on the reaching trajectories were performed on the *x*, *y*, and *z* data from the index finger marker, except for grip aperture, which was calculated from the data from both markers. The raw 3-D data were filtered using a dual-pass low-bandwidth Butterworth filter (2nd order, 20-Hz cutoff) and were normalized using a cubic spline interpolation into 100 samples (see also Mon-Williams, Tresilian, Coppard, & Carson, 2001; Tresilian, Mon-Williams, Coppard, & Carson, 2005). Velocities were calculated in each cardinal dimension and were used to define the beginning of the movement (Schot, Brenner, & Smeets, 2010). Here, the movement onset was determined when the marker position was sufficiently close (3 cm) to the starting button and the velocity exceeded 5 mm/ms for at least 50 ms.

Trials were rejected when participants started to move before the go signal or if participants moved during no-go trials. One participant was rejected from further analysis in Experiment 1 because he exceeded our no-go failure criterion of 67% correct no-go responses.

Using the position, velocity, and stimulus presentation data, we calculated the following measures: reaction time (time between the flash and movement onset), movement time (time from movement onset until movement offset), grip aperture (3-D distance between the thumb and index finger markers), peak velocity (maximum velocity attained during a movement), time to peak velocity (time from movement onset until the peak velocity was reached), position at passing (distance between the location of the index finger and the nontarget at the moment that the hand passed the vertical position of the middle of the nontarget), and error at passing (the within-subjects error for deviations at passing across repetitions for a condition).

## Results

We performed an initial repeated measures analysis of variance (ANOVA) on the data from Experiment 1 with an extra factor Block (two levels: first and second) and the within-subjects factor Flash Location (three levels: top, right, left). Our analysis showed no significant difference between reaches performed in the first versus the second half of Experiment 1 for all dependent measures (all *p*s > .05). Therefore, the split-half data were collapsed.

## Position at passing

**Fig. 2:**
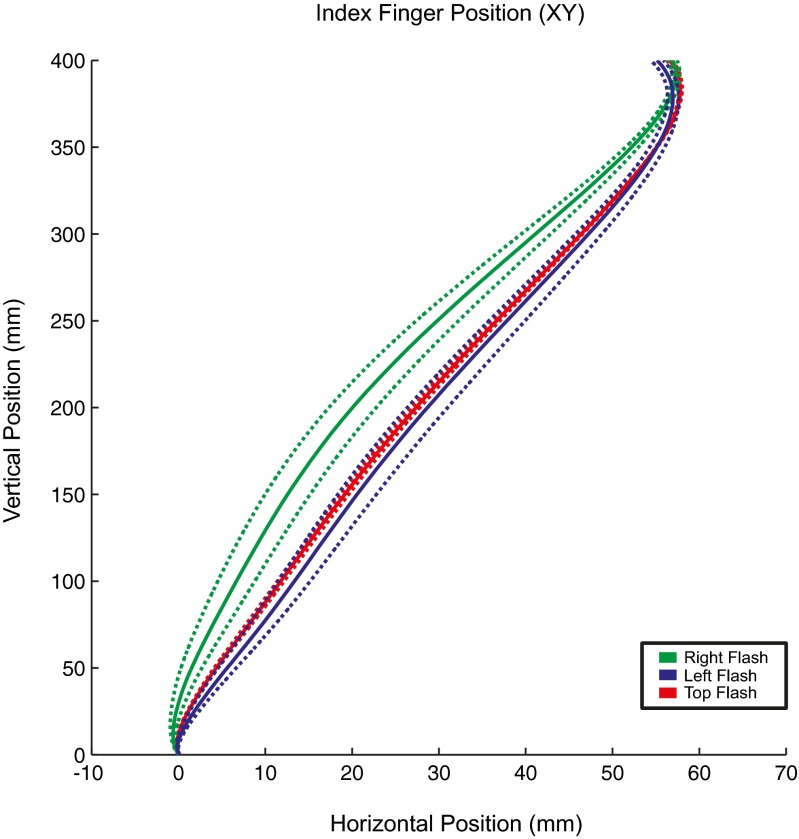
Mean trajectories of the index finger for Experiment 1. Mean movement trajectories were calculated across participants in the *x*, *y* plane. *Solid lines* represent the mean trajectories for reaches, whereas *dotted lines* represent the between-subjects standard errors of the means, which have been corrected for repeated measures by using the Cousineau (2005) method. Line is used to depict the different conditions, in which the left, right, or top obstacle LEDs were flashed. Please note that the endpoint of the reach is near the edge of the target object, which means that half the width of the object and the whole width of the finger “inflate” this endpoint.

**Fig. 3:**
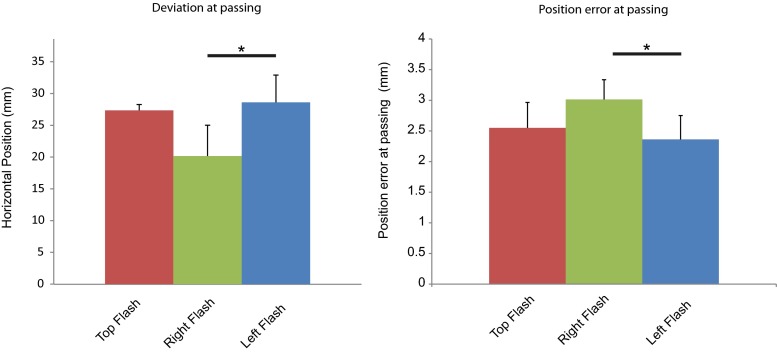
Detailed main effects in Experiment 1. Bar charts show the mean deviation at passing and the mean position error at passing. Error bars show the standard errors of the means. Asterisks denote significant paired *t* tests (Bonferroni-corrected). Line is used to depict conditions in which the left, right, or top obstacle LEDs were flashed.

The results of Experiment 1 are shown in Figure 2. We confirmed that position at passing was significantly affected by the locations of the flashes, *F*(2, 18) = 5.814, *p* = .015, *η*_p_^2^ = .45. The means of the position at passing for the three locations of the flashes were 27.4 mm (±0.9) for the top flash condition, 20.4 mm (±4.9) for the right flash condition, and 28.6 mm (±4.3) for the left flash condition. These data are also displayed in Figure 3. Panel A which shows that when flashes were presented on the right nontarget, participants moved their hand the most to the left, and for flashes on the left, the participants moved their hand the most to the right. Therefore, the participants deviated away from the flashed locations. Further Bonferroni-corrected comparisons revealed that the horizontal position of the hand after right flashes only differed significantly from that under left flashes, *t*(9) = 3.88, *p* = .006, and not from top flashes, *p* > .05.

**Fig. 4:**
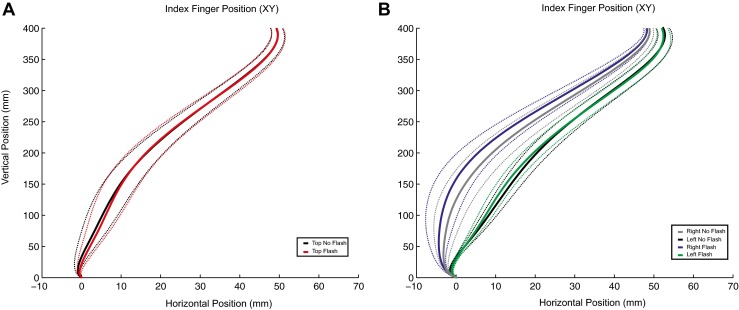
Mean trajectories of the index finger for Experiment 2. Mean movement trajectories were calculated across participants in the *x*, *y* plane. *Solid lines* represent the mean trajectories for reaches, whereas *dotted lines* represent the between-subjects standard errors of the means, which have been corrected for repeated measures by using the Cousineau (2005) method. Panel A shows reaches performed with two obstacles, whereas panel B shows reaches performed with a single obstacle, at either the left or the right of the workspace. In panel A, the trajectories for conditions in which no LEDs or both top obstacle LEDs were flashed are depicted. Panel B shows the results when the right or left top obstacle LEDs were flashed, as well as conditions in which there was no flash with an obstacle on the right or the left.

In Experiment 2, we found a significant interaction between batch (three levels: dual obstacles, single left obstacles, and single right obstacle) and flash (two levels: on and off), *F*(2, 18) = 4.963, *p* = .019, *η*_p_^2^ = .357. Trajectory data from these conditions are displayed in panels A and B of Fig. 4. Panel A shows data from the dual-nontarget setup, whereas panel B shows trajectory data from the single-nontarget setups (on either the left or the right of the workspace). Further Bonferroni-corrected testing revealed that this effect was driven by a significant difference between the flashed and not-flashed right nontarget conditions, *t*(9) = 2.49, *p* = .034. Therefore, participants deviated more away from a right nontarget when it was flashed. Moreover, a flash on the left of the workspace or a flash not limited to a single location (i.e., top) did not affect the reaching trajectories. This was a replication of the results from Experiment 1, as well as confirmation of the nonspatial nature of the top flash cue in Experiment 1.

## Position error at passing

The data of Experiment 1 indicated an effect of flash location on the within-condition variance for horizontal position over trials, or position error at passing: *F*(2, 18) = 13.75, *p <* .001, *η*_p_^2^ = .26. The mean position errors at passing were 2.6 (±0.4) for the top flash condition, 3.0 (±0.3) for the right flash condition, and 2.4 (±0.4) for the left flash condition, which are also displayed Figure 3. Panel B results show that the mean over trial errors was highest when flashes were on the right, whereas it was lowest when the flashes were presented on the left. The intermediate score was for the top flashes. A tandem of Bonferroni-corrected paired *t* tests revealed that the mean error score was higher for the right than for the left, *t*(9) = 2.97, *p* < .05. This implies that participants made more variable movements in the condition in which the most obstructing object in the workspace was flashed.

We did not find any effect of flash location on position errors at passing in Experiment 2.

## Reaction time, movement time, peak velocity

We observed no main effect of flash location on the reaction times, movement times, or peak velocities in either experiment. The fact that reaction times did not differentiate the flash location conditions was expected, because the flashes were used as a general go signal and their individual natures did not necessitate unique response mappings. In addition, participants did not speed up their movements: Peak velocities, times to peak velocity, and movement times were invariant. A separate analysis showed a trend, *p* = .08, toward significance for the effect of flash location on 2-D trajectory lengths (determined by numerical integration of the average spline curve of each condition for each participant). Taken together, this shows that participants moved with the same peak velocity and movement time along curves that were not different in length.

### Grip aperture

We found no effect of flash location on the grip apertures in either experiment. This means that participants did not manipulate the opening between their fingers as part of their avoidance response to the different locations of the flashes coming from the nontargets.

## Discussion

We investigated whether a spatial cue can influence obstacle avoidance. In our experiments, visual cues were go cues for an obstacle avoidance task: Participants had to reach for and grasp a target object while avoiding one or two obstacles. We showed that the avoidance response is tuned by a spatial cue. Specifically, the differences seemed primarily driven by the location of the spatial cue: Only when the right obstacle was flashed (a right-object spatial cue) did participants’ avoidance behavior differ from that to the nonspatial cues. Given that the physical objects were equally obstructing in all conditions, the only thing that could have led to the occurrence of larger avoidance responses was the presence of spatial cues. So, we have now demonstrated the exclusive effect of attention on obstacle avoidance. However, this effect is limited to obstacles that are already more obstructing, because the left-obstacle spatial cue did not have an effect on avoidance movements. The asymmetric avoidance responses observed here, but also previously (Chapman & Goodale, 2008; Dean & Bruwer, 1994; Menger et al., 2012, Menger, Van der Stigchel, & Dijkerman, 2013; Mon-Williams et al., 2001), occur because only the right obstacle interferes more with the transport of the lower limb as the arm extends toward the target object (Menger et al., 2012). Therefore, the hand has to take a more curved trajectory around it. This study further serves to demonstrate that, in terms of the attentional landscape, the spatial location of a more-obstructing object is likely associated with greater activity. We speculate that the subsequent tuning of this activity by a spatial cue is only by virtue of its having a certain level of activity already.

Chapman, Gallivan, Culham, and Goodale (2011) showed that when obstacles interfere with grasp planning, there is top-down modulation in the early visual cortex. Specifically, these authors demonstrated that objects that physically interfere with to-be-performed actions are detected by the contralateral (with respect to the reaching hand) posterior intraparietal sulcus (IPS), which then suppresses the neural representation in early visual cortex areas that is associated with these objects. In addition, Chapman and colleagues (2011) showed that the modulation of visuomotor planning areas by the IPS is dependent on the degree of interference or obstruction afforded by the object. This means that the more the object obstructed movements, the more activity was registered in IPS. In broad strokes, Chapman and colleagues (2011) defined obstacle avoidance along the following lines: Positive neural activity is evoked by physical objects in the visual cortex, and this activity represents a retinotopic map of the workspace. This map can be pictured as a landscape with hills and valleys of activity, both noting locations that are relevant for actions. Movements are drawn toward high-activity regions (hills) in this landscape and are repulsed from low-activity regions (valleys). So, obstacles first will give rise to activity in the landscape and will attract attention. Only when obstacles are tagged by the IPS as interfering with a movement to the target will the activity (hill) be suppressed to a relatively low level (valley). The function of the IPS in this case appears to be to reduce the activity of peaks in a visual attentional landscape, to ultimately have the hand move away from certain objects while it travels toward the goal object.

In our experiments, we offered a spatial cue by flashing LEDs on obstacles. This led to the capture of attention at the obstacle location. This should have served to enhance the activity of peaks in the visual attention landscape associated with the obstacles. Possibly, this enhancement could lead to the obstacle becoming more of an obstacle as measured in motor responses, because the IPS would have already tagged the object as an obstacle at that location. In other words, the obstacle is “marked” by the IPS as a repeller, and attentional capture at the location of the obstacle causes the avoidance system to treat the obstacle as an even stronger repeller.

One caveat is in order, though: In our experiments there was much trial-by-trial consistency in the locations of the targets and nontargets, and this may have led to a static and prebiased attentional landscape (which might be an unfair reflection of real-life dynamic attentional landscapes). Unlike Chapman et al., (2011), we did not use long delays between revealing what objects were obstacles and what were targets, which allowed the landscape to start out neutral and change dynamically over time. The static nature of the landscape and the feedforward nature of motor commands might further reconcile our results with those of most other studies that have used salient distractors, which have shown that distractors can attract trajectories rather than repel them. That is, here it is likely that the extensive experience that participants had with the obstacle setup and the to-be-performed movement ensured that the incoming visual transient from the LED flash was never processed as a target, and therefore was never distracting. This sort of filtering of the incoming visual transient has been described previously in electrophysiological studies (e.g., Ikeda & Hikosaka, 2003).

So, although our results may partly be explained by the experience that participants had with the setup, it is still interesting to see that flashing LEDs can change the weighting of the attentional landscape, as observed through stronger avoidance responses. To wit, a nontarget may prime an additional activation in the attentional landscape that competes with the activation for the target. Top-down processes operate on that competition by “tagging” the obstacle response code. Whether the IPS tag attenuates the obstacle activation to a low hill or valley in the attentional landscape or switches on a different motor-programming module (“avoid” rather than “move to”) is beyond the scope of this article. However, we speculate that our attentional manipulation may have modulated the gain of the IPS response on a trial-by-trial basis; that is, similarly to how Chapman et al. (2011) demonstrated increased IPS activity with increased obstruction, we speculate that attentional capture leads to a more obstructing obstacle, in terms of IPS activity.

To recap, we have found evidence for the effect of stimulation on obstacle avoidance movements. This effect seems to be driven mainly by a spatial cue on a location that obstructs the movement of the arm as it extends toward the target object. We consider that the cue may have changed the attentional distribution across the action-relevant locations in the workspace. Because peaks in the attentional landscape are thought to be intrinsically coupled with motor plans, we observed an effect on hand movements. Specifically, we found that a flash on the more-obstructing object served as a stronger repeller of the heading of the hand.

## References

[CR1] Baldauf D, Deubel H (2010). Attentional landscapes in reaching and grasping. Vision Research.

[CR2] Chang SWC, Abrams RA (2004). Hand movements deviate toward distracters in the absence of response competition. Journal of General Psychology.

[CR3] Chapman CS, Gallivan JP, Culham JC, Goodale MA (2011). Mental blocks: fMRI reveals top-down modulation of early visual cortex when obstacles interfere with grasp planning. Neuropsychologia.

[CR4] Chapman CS, Goodale MA (2008). Missing in action: The effect of obstacle position and size on avoidance while reaching. Experimental Brain Research.

[CR5] Cisek P (2007). Cortical mechanisms of action selection: The affordance competition hypothesis. Philosophical Transactions of the Royal Society B.

[CR6] Cousineau D (2005). Confidence intervals in within-subject designs: A simpler solution to Loftus and Masson’s method. Tutorials in Quantitative Methods for Psychology.

[CR7] Dean J, Bruwer M (1994). Control of human arm movements in two dimensions: Paths and joint control in avoiding simple linear obstacles. Experimental Brain Research.

[CR8] Ikeda T, Hikosaka O (2003). Reward-dependent gain and bias of visual responses in primate superior colliculus. Neuron.

[CR9] Menger R, Dijkerman HC, Van der Stigchel S (2013). The effect of similarity: Non-spatial features modulate obstacle avoidance. PLoS ONE.

[CR10] Menger R, Van der Stigchel S, Dijkerman HC (2012). How obstructing is an obstacle? The influence of starting posture on obstacle avoidance. Acta Psychologica.

[CR11] Menger R, Van der Stigchel S, Dijkerman HC (2013). Outsider interference: No role for motor lateralization in determining the strength of avoidance responses during reaching. Experimental Brain Research.

[CR12] Mon-Williams M, McIntosh RD (2000). A test between two hypotheses and a possible third way for the control of prehension. Experimental Brain Research.

[CR13] Mon-Williams M, Tresilian JR, Coppard VL, Carson RG (2001). The effect of obstacle position on reach-to-grasp movements. Experimental Brain Research.

[CR14] Schot WD, Brenner E, Smeets JBJ (2010). Robust movement segmentation by combining multiple sources of information. Journal of Neuroscience Methods.

[CR15] Tipper SP, Howard LA, Jackson SR (1997). Selective reaching to grasp: Evidence for distractor interference effects. Visual Cognition.

[CR16] Tresilian JR, Mon-Williams M, Coppard VL, Carson RG (2005). Developmental changes in the response to obstacles during prehension. Journal of Motor Behavior.

[CR17] Welsh TN (2011). The relationship between attentional capture and deviations in movement trajectories in a selective reaching task. Acta Psychologica.

[CR18] Welsh T, Elliott D (2004). Movement trajectories in the presence of a distracting stimulus: Evidence for a response activation model of selective reaching. Quarterly Journal of Experimental Psychology.

[CR19] Welsh TN, Elliott D, Weeks DJ (1999). Hand deviations toward distractors: Evidence for response competition. Experimental Brain Research.

[CR20] Wood, D. K., Gallivan, J. P., Chapman, C. S., Milne, J. L., Culham, J. C., & Goodale, M. A. (2011). Visual salience dominates early visuomotor competition in reaching behavior. Journal of Vision, 11(10), 16:1–11. doi:10.1167/11.10.16 10.1167/11.10.16 21940762

